# A Vest-Pocket Medical Lexicon

**Published:** 1866-12

**Authors:** 


### BIBLIOGRAPHICAL.

*A Vest-Pocket Medical Lexicon.*—This neat and very convenient little work has been sent us by the publishers, William Wood & Co., New York. It is said to contain "ten or twelve thousand words," and is of such diminutive size and clear type, that in it a great convenience is afforded the student of medicine. Sent by mail, to those wishing, at *seventy-five cents.* One dollar for a copy in Tuck gilt edge binding.

